# Study of PD-1 Inhibitors in Combination with Chemoradiotherapy/Chemotherapy in Patients with Esophageal Squamous Carcinoma

**DOI:** 10.3390/curroncol29050238

**Published:** 2022-04-20

**Authors:** Tianhui Wei, Wenqi Ti, Qingxu Song, Yufeng Cheng

**Affiliations:** Department of Radiation Oncology, Qilu Hospital of Shandong University, Cheeloo College of Medicine, Shandong University, Jinan 250012, China; 17865195206@163.com (T.W.); tiwenqi@163.com (W.T.)

**Keywords:** esophageal squamous cell cancer, chemoradiotherapy, chemotherapy, immunotherapy, programmed cell death protein 1

## Abstract

In this study, we aimed to evaluate the efficacy of PD-1 inhibitors in combination with concurrent CRT/CT for patients with inoperable ESCC in the real world and to find predictors for the efficacy of PD-1 inhibitors. Patients with unresectable ESCC were evaluated at baseline. The clinical data of patients with ESCC who received CRT/CT with or without PD-1 inhibitor were collected and retrospectively reviewed. The objective response rate (ORR), overall survival (OS), and progression-free survival (PFS) were analyzed statistically. A total of 96 patients with ESCC were included. As compared with a control group (*n* = 48), the PFS (6.0 months vs. 5.0 months, *p* = 0.025) and 6-month OS (70.8% vs. 47.9%, *p* < 0.001) were significantly longer in the ICIs group (*n* = 48). There were no significant differences in ORR and 12-month OS between the two groups. In addition, we found that body mass index (BMI) was associated with PFS (HR 0.85, 95% CI 0.76–0.95, and *p* = 0.004) and OS (HR 0.82, 95% CI 0.69–0.98, and *p* = 0.033) in the ICIs group. PD-1 inhibitors combined with CRT/CT is safe with acceptable complications and improved survival for patients with inoperable ESCC. CRT plus PD-1 inhibitor has superior antitumor efficacy. BMI was positively correlated with the efficacy of PD-1 inhibitors.

## 1. Introduction

The most common histological subtype of esophageal cancer, one of the leading causes of cancer deaths worldwide [1], is esophageal squamous cell carcinoma (ESCC), with the highest incidence in East and Southeast Asia [2]. Definitive CRT is the standard of therapy for patients with inoperable ESCC. Unfortunately, with many patients suffering local recurrences and distant metastases, the prognosis of ESCC remains disappointing [3,4,5]. As a consequence, there is an urgent need for novel antitumor drugs for the treatment of patients with unresectable locally advanced or recurrent/metastatic ESCC.

In this situation, immune checkpoint inhibitors (ICIs) offer new possibilities for the treatment of advanced ESCC cancers. More and more immunotherapeutic evidence indicates that programmed cell death protein 1 (PD-1) inhibitors have shown promising efficacy in advanced ESCC. In recent years, a number of randomized trials have indicated that immunotherapy is a burgeoning new direction for the treatment of ESCC. At the same time, PD-1 inhibitors have been shown to significantly improve overall survival (OS) in ESCC patients [6,7,8,9]. Most of the previous data are from clinical trials and there is a lack of data from real-world applications.

At the same time, finding biomarkers that can effectively predict the efficacy of PD-1 inhibitors is crucial for patients. Currently, PD-L1 expression is the main biomarker of outcome, and patients with PD-L1-positive tumors have better objective response rates. However, PD-L1 staining cannot be used to accurately predict the effect of PD-1 inhibitors due to low predictive accuracy and dynamic changes. In some trials, some patients with PD-L1-negative tumors have also shown clinical responses to PD-1 inhibitors, with tumor regression or disease stabilization, and in some research, BMI has been shown to be related to the efficacy of PD-1 inhibitors [10,11]. Meanwhile, glucocorticoid is used to reduce the side effects of chemotherapy, but it is not very clear how it affects PD1 efficacy.

In this retrospective study, we investigated the efficacy of PD-1 inhibitors plus CRT/CT applied to patients with unresectable locally advanced or recurrent/metastatic ESCC. Therefore, we studied the influences of BMI and glucocorticoid application on the efficacy of PD-1 inhibitors.

## 2. Materials and Methods

### 2.1. Patient Selection

We retrospectively reviewed the medical records of ESCC patients at the Qilu Hospital of Shandong University between June 2018 and June 2021; a total of 628 patients were considered to be unresectable locally advanced or recurrent/metastatic ESCC. After 1:1 PSM, 96 patients with pathologically diagnosed ESCC were selected. All these patients had been histologically confirmed to have primary esophageal squamous cell carcinoma. All patients were first-line treatment failures at the time of enrolment. Moreover, patients were considered to be inoperable patients for the following reasons: (1) unresectable primary tumor, (2) advanced age or poor general health, (3) refusal of surgery, (4) distant metastases and local recurrence, or (5) cervical esophageal cancer. Furthermore, all patients underwent endoscopy/surgery with biopsy, computed tomography scan, and positron emission tomography-computed tomography scan as baseline studies for being evaluated by staging assessment. The clinical TNM stage was based on the 8th American Joint Committee on Cancer (AJCC) TNM staging system and the classification system of Tio et al. [12]. All patients in this study had received more than one course of CRT/CT or CRT/CT plus ICIs. The exclusion criteria were as follows: (1) Eastern Cooperative Oncology Group scoring (ECOG) ≥2, (2) patients with active multiple primary cancers, (3) Patients undergoing ICIs + RCT/CT or RCT/CT followed by surgery.

This study met the standards of the Declaration of Helsinki and was approved by the Ethics Committee of the Qilu Hospital.

### 2.2. Treatment

In the control group, for patients in generally favorable health conditions, chemotherapy consisted of platinum in combination with paclitaxel every 3 weeks (Q3W). The most commonly prescribed dose of radiation was 50.4–60 Gy in 28–30 fractions. While for patients in generally poor health, a single drug (e.g., paclitaxel, platinum, S-1, etc.) was a more common choice. In the ICIs group, the treatment regimen was a PD-1 inhibitor combined with concurrent CRT/CT. The types of PD-1 inhibitors are camrelizumab, tislelizumab, or sintilimab (200 mg i.v. Q3W). Patients received salvage therapy after disease progression.

### 2.3. Outcome Measures

The treatment was evaluated after 2 sessions. During the treatment, imaging evaluation used the Response Evaluation Criteria in Solid Tumors (RECIST) 1.1 standard. A bone scan was also performed if necessary. The primary endpoint of the study was progression-free survival (PFS), defined as the time from the start of CRT/CT to the first documented disease progression, death from any cause, or interruption of follow-up. Secondary endpoints included objective response rate (ORR), overall survival (OS), and safety. Overall survival (OS) was defined as the time from treatment initiation to death of any cause and objective response rate (ORR) was the percentage of patients who achieved a complete response (CR) or partial response (PR). Meanwhile, acute and late toxicities were assessed according to the Common Terminology Criteria for Adverse Events (CTCAE) 4.0.

### 2.4. Statistical Analysis

Propensity score matching (PSM) was used to adjust for unbalanced covariates at baseline, including age, gender, ECOG score, tumor location, endoscopic classification, stage of disease, and concurrent therapy. One-to-one matching is done by matching nearest neighbors for propensity scores. The caliper width was set at 0.05 of the standard deviation of the propensity score. Continuous variables are reported as mean ± SD and categorical variables are reported as rates with 95% confidence interval (CI). The Kaplan–Meier method was used to estimate PFS, OS, and ORR in the ICIs and control groups. Multivariate Cox proportional hazards models were used to compare PFS and OS. All tests were two-sided tests and the threshold for statistical significance was set at *p* < 0.05. Analyses were performed using SPSS 26.0.

## 3. Results

### 3.1. Patient Characteristics

A total of 628 esophageal cancer patients were treated in our hospital from June 2018 to June 2021. In this cohort, 74 patients received PD-1 inhibitors. After 1:1 PSM, 96 patients are divided into the ICIs group and the control group. Each group consists of 48 patients. (Among the 96 patients, 48 vs. 48 patients were included in the ICIs group vs. control group, respectively.). The baseline characteristics of patients are listed in Table 1. In the whole cohort, 85 (88.5%) of the patients were male and the majority of them (90.6%) had thoracic tumors. The BMI followed a normal distribution with a mean of 21.2 ± 0.4 kg/m2 (range of 14.98–30.19). The endoscopic classification was summarized as protruding (60.4%), ulcerative (30.2%), and constrictive (9.4%). Most patients enrolled in this study had advanced ESCC; 9 patients had cervical ESCC (7 in the ICIs group vs. 2 in the control group), while 87 patients had thoracic ESCC (41 vs. 46). These differences are not statistically significant due to PSM. The numbers of patients in stage II, stage III, stage IVA, and IVB were 6(6.3%), 15 (15.6%), 20(20.8%), and 55(57.3%) patients, respectively. All patients selected were regarded as inoperable patients. When patients with recurrent ESCC underwent CRT/CT, their clinical TNM stage was assessed. In this research, 57.3% of patients received CRT and 42.7% of patients received CT. The median follow-up time for surviving patients in the whole cohort was 11.0 months (95% CI 8.3–13.7 months), with 13 months (95% CI 9.2–16.8 months) for the control group and 9 months (95% CI 7.2–10.8 months) for the ICIs group.

### 3.2. Prognostic Analysis

**Figure 1:**
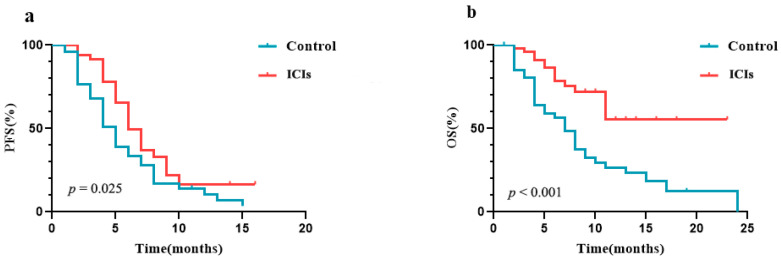
The PFS and OS in the ICIs group and control group. (**a**) The PFS in the ICIs group and control group. (**b**) The OS in the ICIs group and control group.

The ORR was 22.9% (11/48) in the control group and 20.8% (10/48) in the ICIs group. The PFS medians of the control and ICIs groups were 5.0 months (95% CI 4.0–6.0 months) vs. 6.0 months (95% CI 4.9–7.1 months), respectively (Figure 1a). The OS rates at 6 and 12 months in the control group were 47.9% and 16.7%, while those in the ICIs group were 70.8% and 18.8%, respectively (Figure 1b). PD-1 inhibitor was independently associated with PFS (HR 0.52, 95% CI 0.32–0.86, and *p* = 0.011) and OS (HR 0.36, 95% CI 0.20–0.67, *p* < 0.001) (Table 2). Patients receiving concurrent chemoradiotherapy had a better PFS as compared with chemotherapy alone (HR 0.92, 95% CI 0.85–0.99, and *p* = 0.028). However, receiving concurrent chemoradiotherapy did not prolong patients’ OS rates as compared with chemotherapy alone (*p* = 0.25) (Table 2). The results suggest that concurrent chemoradiotherapy had better local control rates than chemotherapy. The effects of age, gender, ECOG, tumor location, endoscopic classification, and stage on PFS and OS were not statistically significant (*p* > 0.05). All patients in the ICIs group completed two cycles of PD-1 inhibitors with CRT/CT. The incidence of treatment-related adverse events (AEs) in the ICIs and control groups were 66.7% (32/48) and 60.4% (29/48) (Table 3).

For grade 3–4 AEs, the incidences in the ICIs and control groups were 22.9% vs. 33.3%. The most frequent grade 3–4 treatment-related AEs in the ICIs and control groups were neutropenia (17.7%) and vomiting (5.2%). Grade 3–4 immune-related AEs were reported in 4 patients (8.4%), including hepatitis in 1 patient (2.1%), myocarditis in 1 patient (2.1%), pneumonitis in 1 patient (2.1%), and colitis in 1 patient (2.1%). No treatment-related deaths occurred.

To further exclude the effect of different treatment modalities on the evaluation of the efficacy of PD-1 inhibitors, different subgroups were set up, namely the RCT subgroup and the CT subgroup. The number of patients in the RCT subgroup was 55 and the number of patients in the CT subgroup was 41 (Table 4). The results showed that the use of PD-1 inhibitors was associated with OS (HR 0.19, 95% CI 0.069–0.509, and *p* = 0.001) and not with PFS (*p* = 0.068) in the RCT subgroup. PD-1 inhibitors were correlated with PFS (HR 0.30, 95% CI 0.127–0.716, and *p* = 0.007) and not with OS (*p* = 0.059) in the CT subgroup.

### 3.3. Influence Factors

To further search for predictors of PD-1 inhibitor efficacy, further relevant studies were conducted in the ICIs group. Age, gender, BMI, ECOG, tumor location, endoscopic classification, stage, and treatment were included, and a multivariate Cox proportional hazards model was constructed after first conducting a univariate Cox model. Higher BMI was independently associated with a longer PFS (HR 0.85, 95% CI 0.76–0.95, and *p* = 0.004) and OS (HR 0.82, 95% CI 0.69–0.98, and *p* = 0.033) in the ICIs group (Table 5). To further validate the findings, the same study was performed in the control group and the results showed that BMI was not associated with OS and PFS in control patients. We did not find strong evidence of an association between PFS/OS and endoscopic classification.

**Figure 2:**
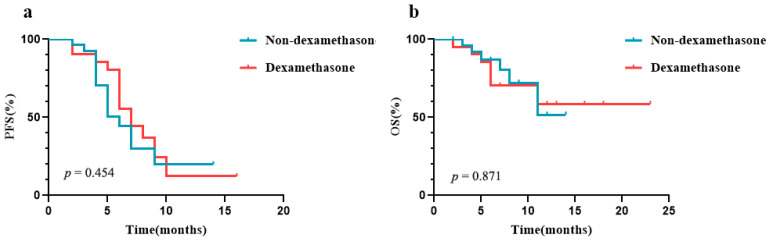
The PFS and OS for patients treated with dexamethasone in the ICIs group. (**a**) The PFS for patients treated with dexamethasone in the ICIs group. (**b**) The OS for patients treated with dexamethasone in the ICIs group.

In the ICIs group (*n* = 48), 21 patients (43.8%) were treated with dexamethasone 5 mg at the initiation of PD-1 inhibitors, while the remaining 27 patients (56.2%) did not use dexamethasone. The Kaplan–Meier curves showed no significant difference in OS (*p* = 0.871) and PFS (*p* = 0.454) due to the use of dexamethasone (Figure 2). The results suggest that the use of 5 mg dexamethasone, when treated with PD-1 inhibitors in combination with chemotherapy, did not affect patients’ PFS or OS rates.

## 4. Discussion

We retrospectively analyzed the efficacy of PD-1 inhibitors in combination with CRT/CT in the real world, including a control group. Eventually, our results indicate that anti-PD-1 immunotherapy in combination with CRT/CT is more effective than CRT/CT for patients with inoperable ESCC. The OS and PFS were significantly prolonged. Moreover, the rates of AEs were similar between the groups.

In our study, CRT/CT plus ICIs has shown promising efficiency and safety in ESCC, which is also consistent with many clinical trials [6,7,13]. After matching for baseline characteristics, PD-1 inhibitor was an independent prognostic factor associated with better OS and PFS. In addition, we found that even in patients with advanced ESCC, CRT had better local control as compared with a CT, which is consistent with previous research findings. Patients with thoracic ESCC account for the majority (90.6%), thus, this study is more applicable to thoracic ESCC. We believe that some of the negative results in the subgroup analysis were due to the small sample size. In addition, the treatment of cervical and thoracic esophageal cancer differs in that radiotherapy is recommended for cervical esophageal cancer and surgery is not the first recommended treatment option. In contrast, surgery is recommended for thoracic esophageal cancer, and there are more treatment options available. Seven patients in the ICIs group in this study had cervical esophageal cancer, as compared with two patients in the control group, with some variability. However, as most of the patients included in the study were stage IVa and IVb, and all were assessed as unresectable ESCC, even those with thoracic esophageal cancer, who had lost the opportunity for surgery and for whom concurrent chemoradiotherapy was the treatment of choice, it can be assumed that the treatment modalities for thoracic and cervical esophageal cancer included in the study were approximately the same. Moreover, after PSM, patients achieved 1:1 matching, and differences at the baseline level were not statistically significant after testing. Few patients experienced grade 3–4 acute or late toxicities and the incidence of AEs was similar in both groups. It illustrates the safety of PD-1 inhibitors in antitumor therapy, while some minor adverse reactions may be overlooked due to inadequate clinical information. Hence, more real-world data are needed to validate the efficacy and safety of PD-1 inhibitors in ESCC.

In addition to what we discussed above, patients with esophageal cancer are usually in a poorer nutritional state than patients with other cancers and the BMI indicator can reflect a patient’s physical condition. Our results show that an elevated BMI is associated with longer PFS and OS in the ICIs group. In previous studies, overweight/obese patients who received PD-1 inhibitors usually had better outcomes [10,11,14]. However, in our study, overweight patients receiving PD-1 inhibitors did not show better outcomes. Obesity-related immune dysregulation creates a favorable environment for increased PD-1/PD-L1 inhibitor efficacy by inducing tumor-intrinsic, microenvironmental, and systemic changes [15]. The BMI scores of the patients in our study were distributed at 20.9 (19.0–23.7) kg/m^2^, mostly at normal BMI levels. Therefore, our results suggest that increasing the BMI of ESCC patients within normal levels (≤25 kg/m^2^) may improve the prognosis. Enhancing nutritional management in ESCC patients may improve the efficacy of PD-1 inhibitors. BMI may be a predictor of the efficacy of PD-1 inhibitors.

Several studies have focused on the relationship between macroscopic tumor type and cancer incidence and prognosis. Kubo et al. previously reported a relationship between macroscopic tumor type as a predictive factor for outcomes in local advanced ESCC [16,17]. In our study, endoscopic tumor type was defined as three types and there was no significant association between endoscopic classification and PFS/OS in both groups and in the whole cohort. Based on this study, endoscopic classification should not be used as a reference to guide treatment decisions. In future studies, larger sample sizes and a more scientific approach to staging may lead to different results.

Glucocorticoids are an important and common treatment in cancer patients. In general, glucocorticoids are thought to be associated with reduced efficacy of PD-1 inhibitors by suppressing the effects of T cells. Arbour et al. found that corticosteroids were significantly associated with decreased PFS and OS in patients with non-small cell lung cancer [18]. However, in this study, receiving 5 mg dexamethasone for 1 day did not influence survival. This is consistent with the results of some studies in which the short-term application of small doses of glucocorticoids did not affect the efficacy of PD-1 inhibitors [18,19,20,21]. Based on our result and the current clinical data, low doses of corticosteroids for a short duration may have little effect on long-term antitumor efficacy.

Our study was limited by its retrospective nature and a relatively small sample size. It was at risk of misclassification bias. There may have been inconsistencies in chemotherapy drugs and radiation dose fractionation regimens. Decisions on treatment options were at the discretion of the treating physician. Nevertheless, our study illustrated the effectiveness of PD-1 inhibitors with real-world data. This study provides clinicians with a reference point for the use of PD-1 inhibitors in ESCC patients. There is a need for further studies to assess the efficacy and safety of PD-1 inhibitors in ESCC.

## 5. Conclusions

In conclusion, this retrospective study shows that PD-1 inhibitors plus CRT/CT is effective and safe for inoperable ESCC. The BMI score was used as a predictor of the efficacy of PD-1 inhibitors, and increased BMI was associated with prolonged PFS and OS. Low doses of corticosteroids for a short duration did not affect the efficacy of PD-1 inhibitors.

## Figures and Tables

**Table 1:** Clinical characteristics of patients.

	ICIs Group (*n* = 48) (*n*, %)	Control Group (*n* = 48) (*n*, %)	Total (*n* = 96) (*n*, %)	*p*
Age				0.682
<65	21 (43.8)	23 (47.9)	44 (45.8)	
≥65	27 (56.3)	25 (52.1)	52 (54.2)	
Gender				0.749
Female	6 (12.5)	5 (10.4)	11 (11.5)	
Male	42 (87.5)	43 (89.6)	85 (88.5)	
BMI	20.8 ± 0.5	21.5 ± 0.5	21.2 ± 0.4	0.475
ECOG				1
0	37 (77.1)	37 (77.1)	74 (77.1)	
1	11 (22.9)	11 (22.9)	22 (22.9)	
Location				0.08
Cervical	7 (14.6)	2 (4.2)	9 (9.4)	
Thoracic	41 (85.4)	46 (95.8)	87 (90.6)	
Classification				0.783
Protruding	30 (62.5)	28 (58.3)	58 (60.4)	
Ulcerative	13 (27.1)	16 (33.3)	29 (30.2)	
Constrictive	5 (10.4)	4 (8.3)	9 (9.4)	
Stage				0.845
II	3 (6.3)	3 (6.3)	6 (6.3)	
III	6 (12.5)	9 (18.8)	15 (15.6)	
IVA	11 (22.9)	9 (18.8)	20 (20.8)	
IVB	28 (58.3)	27 (56.3)	55 (57.3)	
Therapy				0.536
RCT	26 (54.2)	29 (60.4)	55 (57.3)	
CT	22 (45.8)	19 (39.6)	41 (42.7)	

RCT, concurrent chemoradiotherapy; CT, chemotherapy.

**Table 2:** Prognostic factors by multivariate analysis.

	PFS		OS	
	HR (95% CI)	*p*-Value	HR (95% CI)	*p*
Therapy				
RCT	0.48 (0.28–0.81)	0.006	1.33 (0.69–2.58)	0.25
CT	Reference			
Therapy				
ICIs	0.52 (0.32–0.86)	0.011	0.36 (0.20–0.67)	0.001
Non-ICIs	Reference			

RCT, concurrent chemoradiotherapy; CT, chemotherapy; ICIs, immune checkpoint inhibitors.

**Table 3:** Adverse events during therapy.

	ICIs Group (*n*, %)	Control Group (*n*, %)
Hypothyroidism	5 (10.4)	0 (0.0)
Elevated transaminase	5 (10.4)	6 (12.5)
Leukopenia	15 (31.3)	17 (35.4)
Thrombocytopenia	0 (0.0)	1 (2.1)
Anemia	1 (2.1)	0 (0.0)
Fatigue	1 (2.1)	0 (0.0)
Pneumonitis	1 (2.1)	0 (0.0)
Rash	1 (2.1)	0 (0.0)
Vomiting	1 (2.1)	4 (8.3)
Fever	0 (0.0)	1 (2.1)
Myocarditis	1 (2.1)	0 (0.0)
Diarrhea	1 (2.1)	0 (0.0)

**Table 4:** Subgroups analysis by COX model.

	PFS		OS	
	HR (95% CI)	*p*-Value	HR (95% CI)	*p*
RCT subgroup				
ICIs	0.50 (0.237–1.054)	0.068	0.19 (0.069–0.509)	0.001
Non-ICIs	Reference			
CT subgroup				
ICIs	0.30 (0.127–0.716)	0.007	0.391 (0.148–1.037)	0.059
Non-ICIs	Reference			

RCT, concurrent chemoradiotherapy; CT, chemotherapy; ICIs, immune checkpoint inhibitors.

**Table 5:** Prognostic factors in ICIs group by COX model.

	PFS		OS	
	HR (95% CI)	*p*-Value	HR (95% CI)	*p*-Value
BMI	0.85 (0.76–0.95)	0.004	0.82 (0.69–0.98)	0.033
Classification				
Protruding	0.90 (0.30–2.69)	0.967	0.52 (0.14–1.97)	0.695
Ulcerative	0.83 (0.25–2.78)	0.652	0.38 (0. 08–1.89)	0.975
Constrictive	Reference			

## Data Availability

The dataset used and/or analyzed during the current study are available from the corresponding author on reasonable request.
